# Cognitive and Electrophysiological Correlates of Working Memory Impairments in Neurofibromatosis Type 1

**DOI:** 10.1007/s10803-021-05043-3

**Published:** 2021-05-08

**Authors:** Gorana Pobric, Jason R. Taylor, Hemavathy M. Ramalingam, Emily Pye, Louise Robinson, Grace Vassallo, JeYoung Jung, Misty Bhandary, Karolina Szumanska-Ryt, Louise Theodosiou, D. Gareth Evans, Judith Eelloo, Emma Burkitt-Wright, Johan Hulleman, Jonathan Green, Shruti Garg

**Affiliations:** 1 Division of Neuroscience & Experimental Psychology, School of Biological Sciences, Faculty of Biology, Medicine and Health, University of Manchester, Room 3.310 Jean McFarlane Building, Oxford Road, Manchester, M13 9WL UK; 2 Child & Adolescent Mental Health Services, Royal Manchester Children’s Hospital, Central Manchester University Hospitals NHS Foundation Trust, Manchester Academic Health Sciences Centre, Manchester, UK; 3 Nationally Commissioned Complex NF1 Service, Central Manchester University Hospitals NHS Foundation Trust, Manchester Academic Health Sciences Centre, Manchester, UK; 4 School of Psychology, Precision Imaging Beacon, University of Nottingham, Nottingham, UK; 5 Faculty of Biology, Division of Evolution and Genomic Sciences, Manchester Centre for Genomic Medicine, Medicine and Health, North West Genomics Hub, University of Manchester, Manchester, UK

**Keywords:** Neurofibromatosis 1, Working memory, N-back task, EEG, P300

## Abstract

Neurofibromatosis 1 (NF1) is a single gene disorder associated with working Memory (WM) impairments. The aim of this study was to investigate P300 event-related potential (ERP) associated with WM in NF1. Sixteen adolescents with NF1 were compared with controls on measures of WM and EEG was recorded during a WM nback task. The NF1 group showed poorer performance on measures of WM as compared to the control group. No group differences were observed in P300 amplitude at Pz, but P300 latency was shorter in the NF1 group. Topographic analyses of P300 amplitude showed group differences indicating neural processing differences in the NF1 group relative to controls, which possibly contribute to the cognitive deficits seen in this population.

**Supplementary Information:**

The online version contains supplementary material available at 10.1007/s10803-021-05043-3.

## Introduction

Neurofibromatosis 1 (NF1) is a common single-gene autosomal dominant neurodevelopmental disorder with birth incidence of 1:2700 (Evans, 2010). Although well-known for its cutaneous manifestations, morbidity in NF1 children often results from cognitive, social and behavioural difficulties (Garg, 2013; Lehtonen et al. 2013) which impacts significantly on academic achievement and quality of life. NF1 is commonly associated with Attention Deficit Hyperactivity Disorder (ADHD) in about 40%–50% (Garg et al. 2013; Mautner et al. 2002) and Autism Spectrum Disorder in 25% of the paediatric population. For a substantial majority there is a generalised neuropsychological impairment, but certain areas of learning may also be differentially affected (Hyman et al. 2005; Lehtonen, 2015). Impairments are seen in all aspects of executive function (Plasschaert et al. 2016) including attention (Huijbregts et al. 2010; Isenberg et al. 2013), cognitive flexibility (Roy, 2014), and planning (Galasso, 2014) but visuo-spatial Working Memory (WM) impairment is considered one of the hallmark features of NF1 (Van Eylen, 2017).

WM can be defined as the ability to hold and manipulate information in the mind in service of ongoing cognitive activity (Baddeley & Hitch, 1974). These ‘temporary memory’ abilities play an important underlying role in acquisition of complex skills during development and are strongly associated with academic functioning including in mathematics, literacy, and language comprehension (Gathercole & Alloway, 2006). The functional capacity of working memory steadily improves through childhood until mid-adolescence and by age of 15 years, levels close to that of adults are reached. In the general population, WM impairments are characteristic of neurodevelopmental disorders (Habib et al. 2019) such as ASD and ADHD but also serious mental illnesses such as depression and schizophrenia (Lee & Park, 2005). Further, the developmental trajectories of cognitive maturation processes that support WM have been shown to be delayed in ASD (Luna et al. 2007) and ADHD (Ramos et al. 2020).

Electroencephalography (EEG) has been widely used to investigate the neurophysiological underpinning of WM in typical populations, particularly the event-related potential (ERP) P300 component amplitude and latency (Gevins & Cutillo, 1993). The P300 is a distinctive large positive wave that peaks approximately 300 ms post stimulus up to 800 ms or more with maximal amplitude over the parietal midline area. For studies of working memory, the *n-back task* is a frequently used paradigm to elicit the P300. The n-back task has two distinct sub components- a working memory updating/rehearsal component and a second component involving comparison of the probe stimulus to the memory set. In order to successfully perform this task, individuals need to maintain information of a given stimulus for one or more successive trials during which they perform a matching task to compare a new stimulus to one currently held in working memory. P300 amplitude has been related to attention allocation and memory updating, and its latency can be taken to reflect information processing time (Polich, 2007). In healthy participants, the P300 amplitude reduces with increasing memory load (McEvoy et al. 1998), suggesting reallocation of processing capacity from the comparison subtask to memory maintenance (Watter et al. 2001). Further, topographic distribution of P300 may be used to infer information about underlying neural generators (Polich, 1997).

As a single gene disorder that impacts memory, NF1 provides a useful model to understand its underlying biological mechanisms; and WM impairments in particular have been characterized in *Nf1*^*(*±*)*^ mouse models. Pathogenic variants of the NF1 gene and consequent increase in Ras/MAPKinase signaling result in GABAergic overactivity. This increased GABAergic inhibition disrupts corticostriatal activity and contributes to WM impairments in NF1 (Shilyansky, 2010). In a functional neuroimaging (fMRI) study using a spatial capacity task, Ibrahim, (2017) found significant hypoactivation of the left dorsolateral prefrontal cortex (DLPFC) and right parietal cortex during working memory tasks in adults with NF1 as compared to controls. Comparing high vs low memory load conditions**,** the NF1 group showed a more diffuse pattern of brain activation possibly suggestive of less efficient pattern of neural activity (Ibrahim et al. 2017). Overall, the handful of studies which have investigated the neural underpinnings of WM impairments in NF1 using functional neuroimaging suggest altered neural activity including hypoactivation of key working memory areas, abnormal activation between parietal regions and deficient deactivation of default mode network during task (Ibrahim et al. 2017; Shilyansky et al. 2010; Violante, 2012).

Against this background we report the first study to use ERPs to investigate WM at a cognitive and neural level in a pediatric NF1 population matched to healthy controls. More specifically, the focus here is on the P300 component to understand the neural activity related to attentional and WM processes. We sought to elucidate whether the P300 amplitude, latency, and topographic distribution in NF1 differs from typically developing controls. Based on findings in other disorders associated with WM impairments, our aims were to investigate whether the P300 differed between NF1 and control groups in terms of (i) amplitude and latency, (ii) sensitivity to changes in working memory load, and (iii) topographic distribution over the scalp. Further, for any group differences found, we sought to relate individual differences in P300 measures to individual differences in WM performance and more general measures of cognition.

## Methods

### Design and Participants

The NF1 sample was recruited via the cohort of patients at the Manchester Centre for Genomic Medicine and through NF charities newsletters and social media pages. Participants were children aged 12–17 years meeting the National Institute of Health (National Institutes of Health Consensus Development Conference, 1988) diagnostic criteria for NF1. The exclusion criteria were (i) history of epilepsy, (ii) ongoing active treatment for any NF1 related complications (such as chemotherapy for optic glioma), or (iii) other clinically significant unrelated illness. Participants on neuroleptic or stimulant medications were not excluded from this study. All patients that met the eligibility criteria for the study were sent study information packs and were invited to return their indication of interest forms if they wished to participate in the study. A community age and sex matched control sample was recruited through advertisements in the institutional newsletters and approaching local schools in the area. Inclusion criteria for the control group were (i) children aged 12–17 years and (ii) absence of any pre—existing medical conditions or neurodevelopmental disorders.

### Procedure

Once the research team received the ‘Indication of Interest’ form, contact was made with the potential participant family to establish eligibility over the telephone and book the study visit. Oral and written consent from parents and assent from children (where developmentally appropriate) was obtained. Parent rated measure of child adaptive functioning was obtained as a proxy for developmental level. The behavioural assessments were first administered in the lab followed by the EEG session. Clinical notes of the participants were reviewed to confirm comorbid diagnoses of neurodevelopmental disorders.

## Measures

### Behavioural Assessments

#### Working Memory Adaptive N-back Tasks

**Fig. 1:**
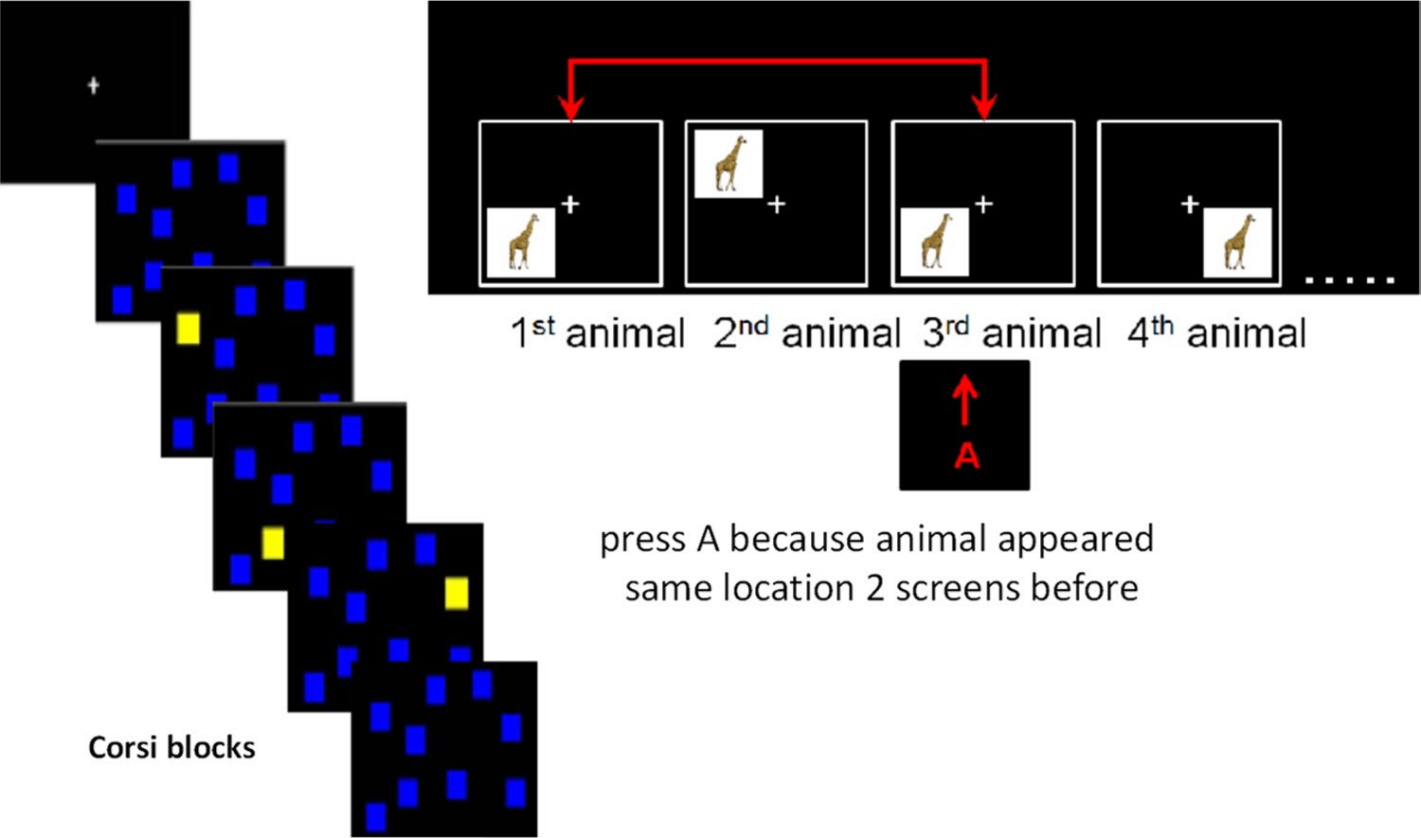
Schematic representation of the Corsi block and the visuospatial n-back tasks

Visuospatial and auditory adaptive n-back tasks were used to assess WM (Conway et al. 2005; Kane & Engle, 2002) in the lab before the EEG session. Based on our pilot results, we used a simplified version of a task designed for testing WM in healthy adults described in previous studies (Jaeggi et al. 2009). The simplified visuospatial stimuli consisted of pictures of animals appearing at one of 4 different loci (instead of 9 in the original task) spaced equally and symmetrically around a constantly present white fixation cross in the centre of a black screen (monitor-to-eyes distance 56 cm). For the auditory task, the verbal material comprised eight aurally presented English consonants (c, g, h, k, p, q, t, w) spoken by a female voice. For both N-back tasks, the stimuli lasted for 1000 ms with an interstimulus interval of 2500 ms (Fig. 1). Participants were instructed to respond as quickly and accurately as possible whenever the current stimulus was the same as the one presented N positions back in the sequence (N depending on the load level, that is, 1, 2, 3; see Fig. 1). The task was adaptive: if the performance was 90% or above in one particular n-back block, the level of the next block was increased by one. If the performance was 70% or below in one particular n-back block, the level of the next round was decreased by one. Otherwise, the level for the next block stayed the same. After two blocks with unchanged n-back level, the experiment was terminated. Within each block there were 20 critical screens plus the additional screens at the start of the sequence needed to create the particular n-back level. (i.e. the first two screens cannot contain a target in a 2-back task). Six out of the 20 screens contained a target and the other 14 screens were non-targets. No responses were required for non-targets. Performance was assessed via two dependent variables– mean n-back (the average n-back level reached) and response times (RT) for target presses.

#### Corsi Blocks Task

The Corsi Blocks Task was used as an index of visual short-term memory (Lezak, 1995). Originally developed by Corsi (1972), this task consists of nine cubes mounted on a board (Corsi, 1972). The examiner taps a sequence of blocks, which the participant has to repeat subsequently in the correct sequential order. By increasing the length of the sequences, the capacity of the visuospatial short-term memory can be measured. Participants were asked to complete a computerised version of the task using PEBL software (Mueller & Piper, 2014). Flash time was set at 500 ms, with an Inter-Stimulus-Interval of 1000 ms. When two sequences were incorrectly repeated, the task was terminated. The test measured the mean length of the largest two correctly remembered sequences. The understanding of instructions and tasks was verified with 3 practice trials which were three blocks long.

#### The Test of Everyday Attention for Children (TEA-Ch)

This is a standardized assessment measure for attention in children and adolescents. Four subtests of the TEA-Ch (Manly et al. 2001) were used including the Sky Search, Score, Creature Counting, and ‘Sky Search Dual Task’ to measure focussed attention, sustained attention, attentional switching, and dual task attention respectively. Raw scores for each sub-test were converted into age and gender corrected scaled scores based on standardised tables provided in the TEA-Ch manual.

#### Digit Span Forward/Backwards

Working memory capacity was assessed Digit span forward/backward subtest of the Wechsler Intelligence Scale for Children – Fourth edition (WISC-IV) (Grizzle 2011). Age-scaled scores were used for the analyses.

#### Vineland Adaptive Behaviour Scale (VABS-III)

Parents completed the Vineland which is a measure to assess child adaptive behaviour in communication, socialization and daily living skills domain. Standardized age equivalent overall functioning was computed and is expressed as Adaptive Behaviour Composite score (Hill et al. 2017).

### EEG

#### N-Back Task

The task consisted of a fixed order (non-adaptive) of four blocks: 1-back, 2-back, 2-back, 1-back. Stimuli were visually presented single letters. Each experimental block consisted of 100 trials, 25 of which were targets (same letter as n trials back). Targets were roughly evenly distributed across each block and only rarely occurred consecutively (6 instances in 2-back blocks). Stimuli that would be considered a target in the other n-back condition (i.e., 2-back target in 1-back block, or 1-back target in 2-back block) were never presented. On each trial, a fixation cross (‘ + ’) was presented in the centre of the screen for 2000 ms (+ / − random jitter of up to 100 ms in 17 ms steps), followed by a single uppercase letter in the centre of the screen for 500 ms. Mouse-click responses were allowed for 2000 ms following stimulus onset (the cursor was not visible on screen). No feedback was given during experimental trials. Stimuli were presented in a light grey font on a black background.

#### EEG Data Collection & Data Pre-Processing

EEG data were recorded with a 64-electrode ActiveTwo system (BioSemi, Amsterdam, Netherlands; 64 EEG channels plus HEOG, VEOG, and mastoids, all sampled at 512 Hz). Data analyses were conducted using Matlab (r2012a) and SPM12 (version 7487; https://www.fil.ion.ucl.ac.uk/spm/) (Litvak, 2011) with custom functions (https://www.github.com/jason-taylor) calling several functions from EEGLAB (version 13.6.5b; https://sccn.ucsd.edu/wiki/EEGLAB) (Delorme & Makeig, 2004) and FieldTrip (http://www.fieldtriptoolbox.org/) (Oostenveld et al. 2011). A common pre-processing pipeline was applied to all data: Continuous EEG data were re-referenced to averaged mastoids, down-sampled to 200 Hz, high-pass (0.1 Hz), low-pass (120 Hz), and notch (48-52 Hz) filtered, before epoching (-600 to 1400 ms relative to stimulus onset). Independent component analysis (ICA) was used to identify blink- and eye movement-related artefacts. 32 ICA components were extracted from the 64 EEG channels (only) using EEGLAB’s ‘runica’ function (with temporal extension option). A temporal correlation was computed between each component’s time-course and VEOG and HEOG channel data (all signals filtered between 1 and 20 Hz before correlations were computed). Spatial correlations were computed between each component’s channel weights and the topography of that participant’s average blink (blink events detected automatically using SPM12′s ‘eyeblink’ artefact routine; epochs extracted from -100 to 300 ms around blink events; epochs averaged; blink topography defined as time-window average from 50-250 ms on all EEG channels). For both temporal and spatial correlations, z-scores were computed for each IC’s correlation by subtracting the average of all correlations and dividing by the standard deviation of all correlations. The resulting z-scores then index not simply the magnitude of the correlation but how unusual it is relative to other IC-artefact correlations. Components with high (absolute) z-scores (> 2) were identified as ‘suspects’ and their time-courses and channel-weight topographies visually inspected. A channel-weight projection matrix was created to remove components that were confirmed to be related to eye-related artefacts (Controls: *M* = 1.73 ± 0.46, range 1–2 components removed; NF1: *M* = 1.88 ± 0.50, range 1–3 components removed). To reconstruct any noisy channels, a channel-weight interpolation matrix was created using FieldTrip’s ‘channelrepair’ function (Controls: 1.40 ± 0.51, range 1–2 bad channels interpolated; NF1: 2.19 ± 0.83, range 1–4 bad channels interpolated; channel TP7 was persistently bad and interpolated for all participants). These two weight matrices were then applied to the epoched EEG data using SPM12′s ‘montage’ function.

#### P300 ERP Analyses

ICA-cleaned N-back trial data were cropped to -100 to 900 ms relative to stimulus presentation and baseline corrected (-100 to 0 ms), and a 30-Hz low-pass filter was applied. Epochs containing (absolute) values greater than 120uV on EEG channels were rejected. Incorrect trials were also rejected; P300 analyses were conducted on correct-trial data only. Because correct non-target trials outnumbered correct target trials, trial numbers were balanced by selecting non-target trials that were temporally proximal to target trials and rejecting the rest. This resulted in an average of 39.2 ± 10 valid trials per condition (range 17–49) for Controls and 38.5 ± 9 (range 17–48) valid trials remaining (the number of trials did not differ significantly between groups, t < 1, p > 0.8).

To identify a time-window and channel for subsequent between-group analyses, grand averages over all participants (collapsing over group) were computed for 1-back targets and non-targets, and the difference (target–non-target) computed. Consistent with the P300 literature, the peak difference occurred on channel Pz, with the two conditions diverging from about 300 ms. Visual offset ERPs were present from about 720 ms (stimulus duration was 500 ms). Therefore, a time-window of 300-700 ms on channel Pz was chosen for ‘canonical’ P300 for amplitude and latency analyses. Time-window averaged amplitude and fractional area latency (the point at which 50% of the area under the ERP in the time window was reached) were computed.

For topographic analyses, in light of latency differences found in the Pz analysis (see Results), the time window was split into early (300-500 ms) and late (500-700 ms) time windows. Topographic maps of time-window averaged amplitude are presented for both target and non-target stimuli. For statistical analysis, data were extracted from four clusters of electrodes representing left and right frontal and parietal regions (see Fig. 5). Four electrodes (F3, F4, P3, and P4; underscored in the lists below) were taken as the ‘centroids’ of these regions, and data from each was combined with that of its 6 nearest neighbours:

left frontal: AF3, F5, F3, F1, FC5, FC3, FC1.

right frontal: AF4, F6, F4, F2, FC6, FC4, FC2.

left parietal: PO3, P5, P3, P1, CP5, CP3, CP1.

right parietal: PO4, P6, P4, P2, CP6, CP4, CP2.

In the topographic ANOVAs described below, two spatial factors – FP (frontal, parietal) and LR (left, right) – were included.

### Statistical Analyses

Statistical analyses were conducted using SPSS version 25 (IBM Corp 2017). The NF1 and control group were compared on demographic and clinical characteristics using two-sample *t* tests for continuous data and χ^2^ tests for categorical data. For standardized measures including Vineland, Conners, digit span and TEA-Ch age scaled scores were used for analyses using two-sample *t* tests. For the nback, Corsi block, and EEG tasks, Pearson correlation coefficients were computed to explore the effect of age on cognitive performance, RT), and P300 measures. Groups were compared using ANCOVA including age as a covariate if the assumptions of homogeneity of regression were met.

For the ‘canonical’ P300 analyses, time-window (300-700 ms) averaged amplitude on Pz for target stimuli and fractional area latency were submitted to 2 × 2 ANCOVAs with n-back (1- or 2-back) as within subject factor, group as between subject factor, and age as a covariate. ANCOVA models with sex as a factor were run, but no effects of sex were found, and the pattern of results did not change; therefore, models reported in the Results section do not include sex.

For topographic P300 analyses, time-window and channel-set averaged amplitude was used as the dependent variable. Because data from neighbouring time-windows are unlikely to be independent, a separate ANCOVA was conducted in each time-window. Therefore, a 5-way mixed ANCOVA with factors Group (Control, NF1), N-back (1-back, 2-back), Condition (target, non-target), FP (frontal, parietal), and LR (left, right), and covariate age, was conducted in each time-window. To simplify the interpretation of the results, we report and follow up only effects involving Group and spatial factors. A criterion value of α = 0.05 was set for all analyses.

Finally, Pearson’s correlations were performed to investigate associations between P300 amplitude/latency (measures selected based on their significance in ANCOVAs) and overall functional ability (parent-reported ABC scores on Vineland), focussed attention (Sky Search subtest of TeaCh), Conners inattention and hyperactivity, and performance on the auditory n-back task from the behavioural session. A false discovery rate (FDR; Benjamini & Hochberg, 1995) of 10% was applied to each behavioural measure’s set of correlations to correct for false positives whilst remaining sensitive to true positives.

## Results

The total sample consisted of 32 participants with 16 participants each in the NF1 and control groups. The mean age of the NF1 group was 13.02 years (SD 1.65, range 11.25–16.58 years) and control group was 13.33 years (SD 1.61, range 11.33–16.92 years). There were no significant differences between the NF1 and control groups in age (t(30) = -0.540, p = 0.593) or sex (9 males and 7 females in each group). Within the NF1 group, 6 had pre-existing clinical diagnoses- 3 with ADHD + ASD, 1 with ADHD and 2 with ASD. The NF1 mutation was inherited in 7 participants and de novo in 9 participants. Four participants were on methylphenidate medication and 2 were on Melatonin.

### Behavioural Results

Descriptive statistics and t-tests comparing groups on all parent reported measures and child behavioural measures are reported in Table 1. There were statistically significant differences between the NF1 and control group on all parent-reported measures. The NF1 group had higher levels of parent reported inattention/ hyperactivity and overall lower adaptive function on the Vineland. The NF1 group showed significantly poorer performance on the digit span tests. On the TEACh, significant differences were observed on the sustained attention and attentional switching tasks (Score and creature counting tasks respectively).

**Table 1:** Comparison of the NF1 group with the control group on standardized measures of attention, adaptive functioning and working memory

	NF1 (n = 16)	Controls (n = 16)	T test	P value
*Parent reported measures*				
Conners T scores				
Inattention	73.63 (12.92)	48.81 (8.16)	6.49	0.000
Hyperactivity	69.88 (18.53)	49.31 (8.90)	4.00	0.000
Vineland				
Communication	82.68 (18.07)	108.65 (8.05)	−5.23	0.000
Daily living skills	85.20 (16.58)	99.00 (16.97)	−2.29	0.030
Socialisation	81.63 (20.72)	106.50 (9.34)	−4.38	0.000
Adaptive behaviour composite	83.06 (16.58)	103.56 (15.35)	−3.57	0.001
Digit span forward	6.75 (3.02)	10.56 (2.33)	−3.99	0.000
Digit span backward	5.88 (1.31)	9.00 (2.31)	−4.71	0.000
TEACH (age scaled scores)				
Sky search attention	10.5 (3.14)	10.81 (2.04)	−0.33	0.741
Score	9.13 (3.81)	11.44 (2.31)	−2.08	0.047
Creature counting	8.62 (3.28)	11.31 (2.98)	−2.42	0.022
Sky search DT	7.88 (1.63)	8.19 (1.94)	−0.49	0.625

To examine group differences on the nback and Corsi block tasks, the relationship between age and dependent variables in the NF1 and control groups was examined (Supplemental Table S1). There were no significant group differences in the Pearson correlation coefficients of age and mean nback/Corsi block task performance. Both the NF1 and control group performed better on these cognitive tasks with age. On the visuospatial nback task, there was significant group differences with poorer performance in the NF1 group (F(1,28) = 26.726, p < 0.001) but there was no effect of age (F(1,28) = 0.875, p = 0.358). On the auditory n-back, there were significant group differences with poorer performance in the NF1 group (F(1,29) = 32.538, p < 0.001) and significant effect of age F(1,29) = 8.000, p = 0.008. On Corsi task, NF1 showed significantly poorer performance (F(1,29) = 4.959, p = 0.034) with significant effect of age (F(1,29) = 9.941, p = 0.004).

**Fig. 2:**
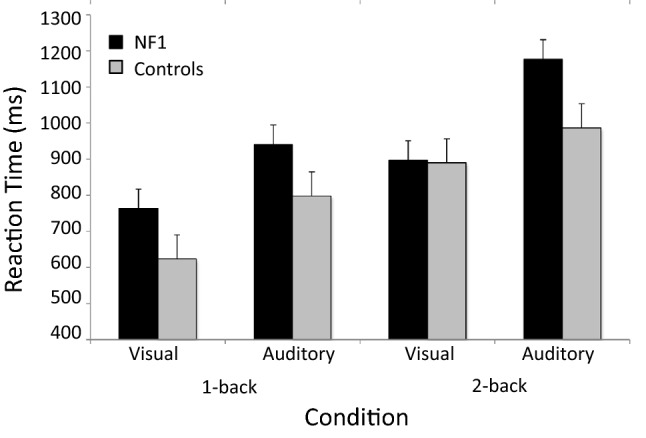
Reaction times of the NF1 and control groups during 1-back and 2-back visuospatial and auditory tasks

**Fig. 3:**
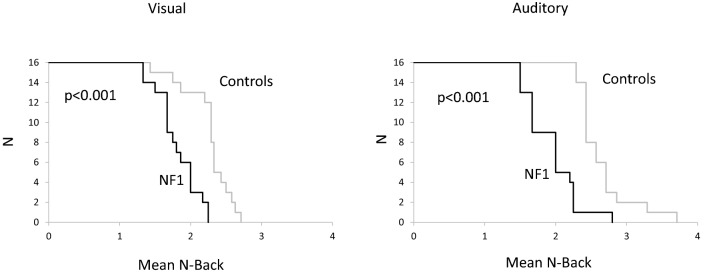
Kaplan Meier curves illustrating mean n-back performance for visual and auditory modality for NF1 and control participants

There was no significant relationship between age and RT in the NF1 group (2back visuospatial task RT *r* = 0.06, p = 0.820, 2-back auditory task RT *r* = 0.08, p = 0.77). However, in the control group RTs were observed to be faster with age (2back visuospatial task r = -0.63, p = 0.009, 2-back auditory task r = -30, p = 0.26). A 2 × 2x2 mixed ANOVAs on RTs for correct responses was performed with Modality (auditory and visual) and n-back level (1 back and 2 back) as within-subject factors and Group (NF1 and controls) as between-subject factor. Age was not included as a covariate given the significant group differences in the Pearson correlation coefficients of age and RT (Supplemental Table 1). The analyses showed the main effect of modality *F*(1, 30) = 18.078, *p* < 0.001, η_p_^2^ = 0.376, with faster RTs for all participants on the visuo-spatial tasks as compared to the auditory task. We also report the main effect of the n-back level *F*(1, 30) = 34.900, *p* < 0.001, η_p_^2^ = 0.538, with faster RTs for 1-back compared to 2 -back blocks. The main effect of Group was approaching significance *F*(1, 30) = 3.820, *p* = 0.06, η_p_^2^ = 0.113 with the NF1 group slower than the controls. All other interactions were not significant p > 0.536 (Figs. 2, 3).

Using the Kaplan–Meier procedure, we explored the behavioural performance in visual and auditory modality on the mean n-back level. The log-rank test revealed statistically significantly differences between groups for both visual modality (log-rank statistic 19.432, two-sided *p* < 0.001) and auditory modality (log-rank statistic 20.457, two-sided p < 0.001).

### EEG Results

#### N-back Task Behavioural Measures

Unlike the behavioural n-back tasks reported above which featured an adaptive staircase procedure, the EEG n-back task was not designed to push participants’ performance to its limits. Rather, it was designed to provide a sufficient number of trials to analyse ERP responses at two levels of memory load (1-back and 2-back) in both participant groups. Indeed, a similar number of correct trials between groups (i.e., no performance difference) is desirable here, since then ERP would not be biased by the number of trials on which they were based. Statistical analysis of task performance is therefore provided only for completeness.

Pearson’s correlations between age and visual n-back task performance (hits–false alarms) were run for each group, and these correlations were compared between groups (see Supplementary Table S2). None of the correlations differed between groups; therefore, age was used as a covariate in the ANCOVA. N-back performance was submitted to a 2 (group: NF1, Controls) × 2 (n-back: 1-back, 2-back) mixed ANCOVA. There was a main effect of n-back (*F*(1,28) = 42.009, *p* < 0.001, *η*_*p*_^*2*^ = 0.600), with better performance in the 1-back (*M* = 85.4 ± 13.9%) than in the 2-back blocks (*M* = 64.1 ± 20.9%); however, there was no main effect of group (*F*(1,28) = 0.234, *p* = 0.632, *η*_*p*_^*2*^ = 0.008) and no interaction between group and n-back (*F*(1,28) = 0.108, *p* = 0.745, *η*_*p*_^*2*^ = 0.004); 1-back: CG: *M* = 87.3 ± 11.1%; NF1: *M* = 83.6 ± 16.2%; 2-back: CG: *M* = 67.2 ± 20.5%; NF1: *M* = 61.2 ± 21.5%). The covariate age showed a significant main effect (*F*(1,28) = 10.745, *p* = 0.003, *η*_*p*_^*2*^ = 0.277), but it did not interact with n-back (*F* = 1.547, *p* = 0.224, *η*_*p*_^*2*^ = 0.052). The correlation between age and n-back performance was positive in both groups and in both n-back levels, indicating that older participants performed better.

For RT in the EEG n-back task, Pearson’s correlations again did not differ between groups (see Supplementary Table S2), so age was covaried in the 2 × 2 ANCOVA. A main effect of n-back was found (*F*(1,28) = 5.607, *p* = 0.025, *η*_*p*_^*2*^ = 0.167), with faster response times in the 1-back (*M* = 598 ± 26 ms) than in the 2-back condition (*M* = 661 ± 25 ms), but there was neither a main effect of group (*F*(1,28) = 0.396, *p* = 0.534, *η*_*p*_^*2*^ = 0.014) nor a group x n-back interaction (*F*(1,28) = 1.183, *p* = 0.286, *η*_*p*_^*2*^ = 0.041). The main effect of age was significant (*F*(1,28) = 4.365, *p* = 0.046, *η*_*p*_^*2*^ = 0.135), but age did not interact with n-back (*F*(1,28) = 0.048, *p* = 0.828, *η*_*p*_^*2*^ = 0.002). Correlations between age and RT were all negative, indicating that older participants responded more quickly.

#### P300 amplitude at Pz

**Fig. 4:**
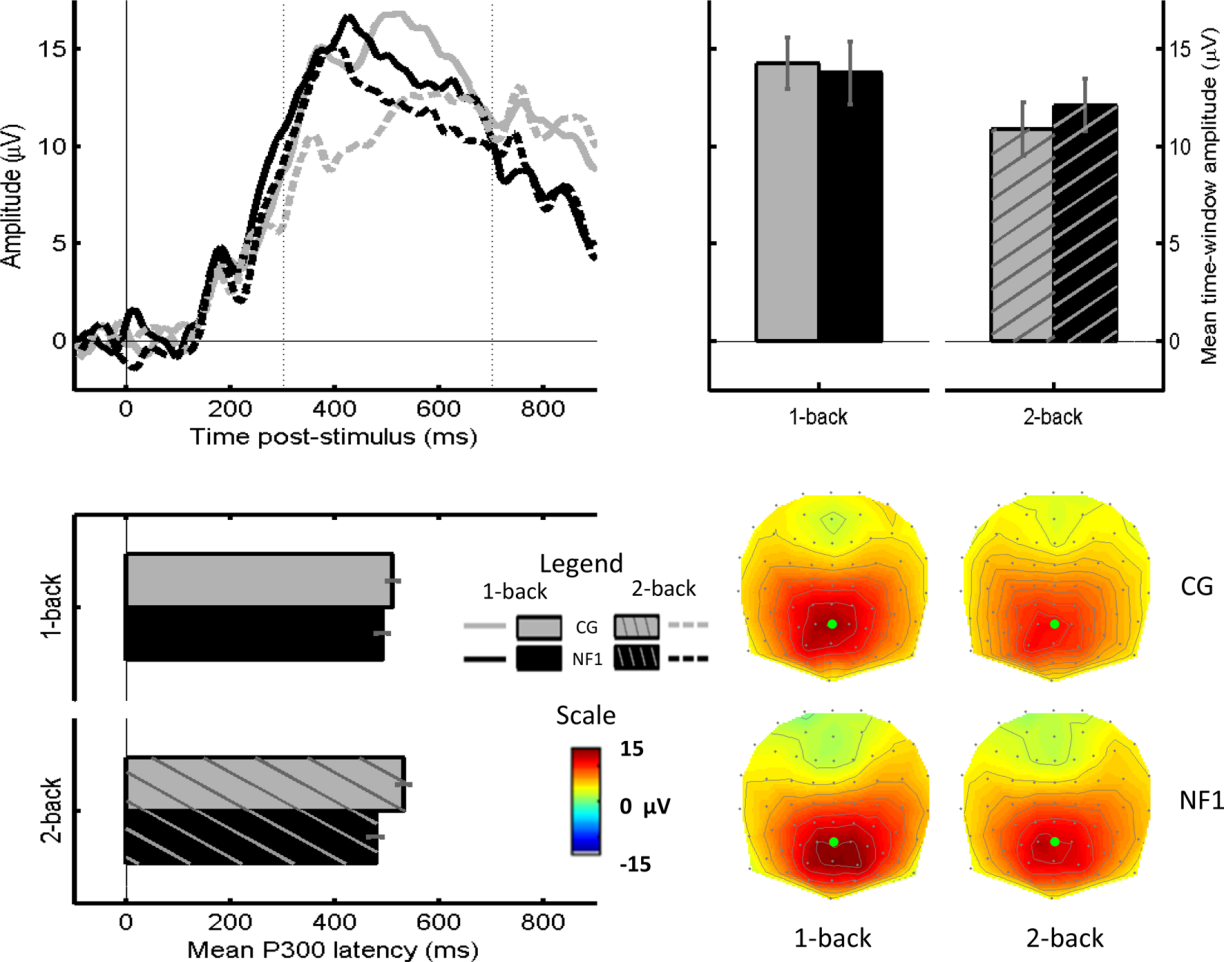
P300 amplitude and latency (at Pz) for targets presented during the 1-back and 2-back tasks. The P300 time-window (300-700 ms) is indicated by vertical dotted lines on the ERP plot. Location of electrode Pz is indicated by a green circle on the topographic plots

Figure 4 shows ERPs from channel Pz and time-window averaged topographies for Controls and NF1 in response to 1-back and 2-back targets, as well as bar charts summarising P300 amplitude and latency in each group. The P300 was maximal on mid-parietal channels (Pz is indicated by green dots on topographies in Fig. 4) in both groups and N-back tasks. ERP time-courses appeared to differ, peaking earlier in NF1 than in Controls, but differentiating between conditions (1-back > 2-back) more in Controls than in NF1. The net result appeared to be no difference in time-window averaged amplitude between the groups, but an apparent difference in latency (NF1 < Controls).

Pearson correlations between age and P300 measures did not differ between groups (see Supplementary Table S3); therefore, age was included as a covariate in all ANCOVA analyses reported below. It is noteworthy that out of 80 age-P300 correlations, only two were significant, which suggests that age did not explain much individual variability in P300 measures overall. Age main effects were non-significant in all ANCOVAs reported below.

In the 2 × 2 Group x N-back mixed ANCOVA on P300 amplitude, a main effect of N-back was found, *F*(1,28) = 11.077, *p* = 0.002, *η*_*p*_^*2*^ = 0.283 – amplitude was larger for 1-back (*M* = 14.002 uV, *SEM* = 1.057) than for 2-back (*M* = 11.497 uV, *SEM* = 0.967). Neither the main effect of Group (*F* < 1, *p* > 0.8) nor the Group x N-back interaction (*F*(1,28) = 1.115, *p* = 0.300) was significant. Age showed no significant main effect or interaction (*F*s < 1, *p*s > 0.3).

#### P300 latency at Pz

The same 2 × 2 Group x N-back ANCOVA was run on P300 fractional area latency. A main effect of Group was found, *F*(1,28) = 6.788, *p* = 0.015, *η*_*p*_^*2*^ = 0.195 with shorter latencies in NF1 (*M* = 485.16 ms, *SEM* = 10.83) than in Controls (*M* = 522.83 ms, *SEM* = 11.19). This group effect was modulated by a significant Group x N-back interaction, *F*(1,29) = 5.794, *p* = 0.023, *η*_*p*_^*2*^ = 0.171. The group difference in latencies was significant in the 2-back task (*t*(29) = 3.082, *p* = 0.004, difference *M* = 54.917 ms, *SEM* = 17.820) but not in the 1-back task (*t*(29) = 1.219, *p* = 0.233, difference *M* = 20.438 ms, *SEM* = 16.768). Additionally, whereas Controls’ P300 latency was significantly shorter in the 1-back than in the 2-back task (*t*(14) = 2.306, p = 0.037, difference *M* = 21.667 ms, *SEM* = 9.394), this difference was not significant for the NF1 latency (*t*(15) = 1.103, *p* = 0.288, difference *M* = 12.813 ms, *SEM* = 11.619). Age showed no significant main effect or interaction (*F*s < 3, *p*s > 0.1).

#### Topographic Analyses

**Fig. 5:**
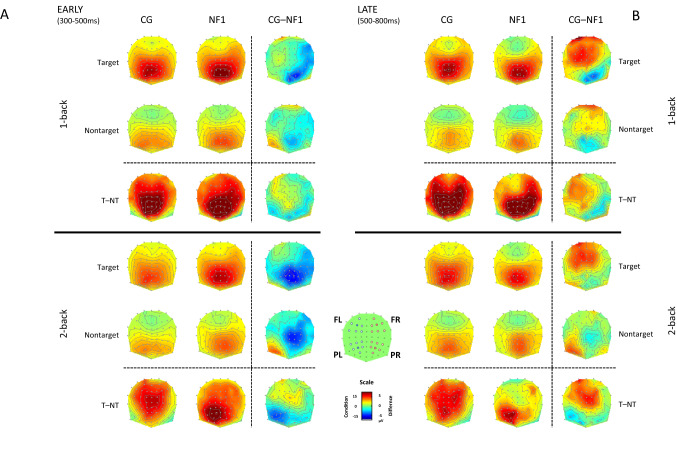
Topographic maps for control (CG), NF1 groups with CG–NF1 difference and condition (rows: target T, non-target NT, T–NT difference), in each N-back task (upper half: 1-back; lower: 2-back) and time-window (A: early, B: late). Differences between groups are most apparent in the condition difference (T–NT, bottom row of each subsection) and group difference (CG–NF1, 3rd column of each sub-section)

Figure 5 shows topographic maps for each group (columns: CG, NF1, CG–NF1 difference) and condition (rows: target, non-target, T–NT difference), in each N-back task (upper half: 1-back; lower: 2-back) and time-window (A: early, B: late). Differences between groups are most apparent in the condition difference (T–NT, bottom row of each sub-section) and group difference (CG–NF1, 3rd column of each sub-section). Across time-windows, the Control target–non-target difference topographies remained relatively stable, with a stronger positivity in the 1-back than in the 2-back task, and with symmetrical frontal positivity in both tasks. By contrast, whilst the NF1 T–NT early 1-back topography looked relatively similar to that of Controls, with only a slight Right > Left asymmetry, the NF1 late 1-back topography showed a strongly asymmetrical (right > left) frontal distribution, and both the early and late 2-back NF1 topographies showed a stronger parietal positivity and weaker frontal positivity than was evident in the Controls. This general pattern was largely supported by the statistical analysis presented below.

### Early Time Window (300-500 ms)

In the early time window, the topographic maps appeared to show a slight Right > Left pattern for NF1, in contrast to a more symmetric pattern for Controls (Fig. 5a, CG–NF1 difference maps in 3rd column, blue patches on right indicating NF1 > CG). This was confirmed by a significant Group x Left–Right (LR) interaction (see Table 2 for statistics). This group difference in hemispheric distribution appeared to be driven by targets in the 1-back task, and by both targets and non-targets in the 2-back task. Further, the mid-parietal response to non-targets in both tasks, and to targets in the 2-back task, appeared stronger in NF1 than in Controls. These observations were confirmed by a significant 5-way interaction, Group x N-back x Condition x Frontal-Parietal (FP) x LR (*p* = 0.044).

**Table 2:** Topographic ANCOVA results (age covaried; only significant and marginal effects involving Group shown)

	*F*	*df*	*p*	*ηp*^*2*^
*Early time window (300-500 ms)*				
Omnibus 5-way ANCOVA				
Group x N-back x Cond x LR x FP	4.450	1,28	.044	.137
Group x Cond x LR x FP	4.016	1,28	.055	.125
Group x LR	7.248	1,28	.012	.206
*1-back* 4-way ANCOVA				
Group x LR	5.075	1,28	.032	.153
*2-back* 4-way ANCOVA				
Group x Cond x LR x FP	7.241	1,28	.012	.205
Group x Cond x FP	4.849	1,28	.036	.148
Group x LR	5.319	1,28	.029	.160
*Frontal 2-back* 3-way ANCOVA				
Group x LR	3.317	1,28	.079	.106
*Parietal 2-back* 3-way ANCOVA				
Group x Condition x LR	2.965	1,28	.096	.096
Group x LR	3.707	1,28	.064	.117
*Late time window (500-700 ms)*				
Omnibus 5-way ANCOVA				
Group x N-back x Cond x LR x FP	5.887	1,28	.022	.174
*1-back* 4-way ANCOVA				
Group x Cond x LR	3.559	1,28	.070	.113
Group x FP	3.093	1,28	.090	.099
*2-back* 4-way ANCOVA				
Group x Cond x FP x LR	3.812	1,28	.061	.120

To follow-up these interactions, we conducted separate 4-way ANCOVAs on the 1-back (Fig. 5a, upper half) and 2-back (Fig. 5a, lower half) task data, respectively. In the 1-back ANCOVA, a significant Group x LR interaction was found. Overall, Controls showed a slight Left > Right pattern, whereas NF1 showed Right > Left (see Fig. 5a); however, between-group t-tests showed no significant differences in either Left or Right amplitude collapsed across Condition and FP (*t*s < 1.5, *p*s > 0.2). When comparing left against right within-groups, Controls showed a marginal Left > Right effect (*t*(14) = 1.869, *p* = 0.083), and NF1 showed a nonsignificant Right > Left effect (*t*(15) = 1.653, *p* = 0.119).

In the 2-back ANCOVA, the Group x LR interaction was also significant, as were the Group x Condition x FP interaction, and the 4-way interaction: Group x Condition x FP x LR. To follow this up, separate 3-way ANCOVAs were conducted on Frontal and Parietal channels, respectively. In the Frontal ANCOVA, there was only a marginal Group x LR interaction (*p* = 0.079), with Controls showing a slight L > R pattern and NF1 showing a slight R > L pattern. In the Parietal ANCOVA, there were marginally significant 2- and 3-way interactions: Group x LR (p = 0.064) and Group x Condition x LR (p = 0.096). Compared to Controls, NF1 showed larger parietal amplitude, particularly for targets, but between-group t-tests produced only trends towards significance (L: *t*(29) = 1.705, *p* = 0.099; R: *t*(29) = 1.929, *p* = 0.064). Within-group t-tests on the T–NT difference comparing left and right parietal sites showed a significant L > R hemispheric difference for NF1, *t*(15) = 3.174, *p* = 0.006, but no hemispheric difference for Controls, *t* < 1 *p* > 0.4.

### Late Time Window (500-700 ms)

In the late time window, Controls appeared to show a larger frontal positivity in response to targets in both 1- and 2-back tasks, particularly at left frontal sites (Fig. 5b, 3rd column CG–NF1 difference maps, red left-frontal patches). This was confirmed by a significant 5-way interaction (Group x N-back x Condition x LR x AP; *p* = 0.022). As in the early time window, this interaction was followed up with separate 4-way ANCOVAs for 1-back (Fig. 5b upper half) and 2-back (Fig. 5b lower half) tasks. In the 1-back ANCOVA, a marginal 3-way Group x Condition x LR interaction (*p* = 0.070) and a marginal 2-way Group x FP interaction (*p* = 0.090) were found. These effects were likely driven by the relative left distribution of the larger positivity for Controls than for NF1, particularly for targets, as evident in the CG–NF1 topography of the difference T–NT. However, no between-group differences were significant for either conditions (target, non-target) or the difference (target–nontarget) in the left frontal region. Within-group t-tests were computed on the T–NT difference to compare left and right frontal sites: a significant Right > Left hemispheric difference was found for NF1, *t*(15) = 3.454, *p* = 0.004, but there was no difference for Controls, *t* < 1 *p* > 0.5 (see Fig. 5b, T–NT difference, 3rd row).

In the 2-back ANCOVA, the 4-way interaction was marginally significant: Group x Condition x FP x LR (*p* = 0.061). The group difference of condition differences (CG–NF1 on T–NT in Fig. 5b, bottom half, 3rd row) topography seems to indicate that Controls show a larger left-frontal positivity (as in the 1-back task), whereas NF1 show a larger left parietal positivity. Between-groups t-tests showed a marginally significant CG > NF1 difference for targets in the left-frontal region (*t*(29) = 1.844, *p* = 0.075); all other contrasts for conditions or the target-non-target difference were non-significant. Within-group t-tests were conducted on the target-non-target difference to compare Left and Right in frontal (as in the 1-back) and parietal regions. A significant parietal Left > Right hemispheric difference was found for NF1, *t*(15) = 3.214, *p* = 0.006 (Fig. 5b lower half, bottom row, T–NT); no such difference was found for Controls (*t*(14) = 1.581, *p* = 0.136), nor was any hemispheric difference were found at frontal sites for either group (*t*s < 1, *p*s > 0.4).

#### Correlations Between P300 Measures and Behavioural Assessments

To relate individual differences in P300 measures and behavioural measures of cognitive function, correlations were run with scores on the Vineland ABC (reflecting overall adaptive functioning), Sky Search (reflecting selective attention), Conners inattention and hyperactivity, and mean n-back on the Auditory N-back task (reflecting working memory capacity, measured independently of EEG). Given the topographic ANCOVA results above, P300 amplitude of the difference (Target – Non-target) was extracted for the following time-window/condition/spatial location combinations: (i) early 2-back left parietal, (ii) late 1-back left frontal, (iii) late 2-back left frontal, and (iv) late 2-back left parietal. Given the group differences in latency found above, 2-back target latency (FAL) at Pz was also included. Correlations are reported in Table 3. A false discovery rate (FDR; Benjamini & Hochberg, 1995) of 10% was applied to each behavioural measure’s set of correlations (n = 10) to correct for false positives whilst remaining sensitive to true positives.

**Table 3:** Correlations between P300 effects and behavioural measures

		P300 amplitude				P300 latency
		Early 2-back Left Parietal	Late 1-back Left Frontal	Late 2-back Left Parietal	Late 2-back Left Frontal	Full time window 2-back targets Pz
Vineland ABC	CG	−.51 (.049*)	−.25 (.373)	−.58 (.022**)	-.61 (.015**)	+ .28 (.316)
	NF1	−.25 (.374)	** + .11 (.697) [.048†]**	−.12 (.661)	**−.03 (.911) [.002†]**	+ .04 (.876)
Sky Search	CG	−.01 (.983)	−.37 (.178)	−.01 (.984)	−.24 (.389)	+ .12 (.669)
	NF1	−.11 (.688)	−.18 (.496)	−.54 (.031**)	**−.46 (.075 ~)**	−.63 (.009**)
Conners IN	CG	+ .35 (.199)	−.39 (.167)	+ .33 (.255)	+ .13 (.656)	−.33 (.230)
	NF1	+ .38 (.152)	−.08 (.777)	+ .39 (.133)	+ .34 (.201)	+ .040 (.883)
Conners HY	CG	+ .32 (.253)	−.08 (.776)	+ .39 (.147)	+ .31 (.261)	−.33 (.226)
	NF1	−.19 (.482)	+ .13 (.636)	−.19 (.475)	−.27 (.305)	−.02 (.946)
Auditory N-back	CG	+ .18 (.520)	**−.47 (.077 ~)**	+ .39 (.156)	+ .04 (.888)	−.07 (.810)
	NF1	+ .07 (.804)	−.05 (.847)	−.19 (.492)	+ .19 (.480)	−.27 (.322)

P300 measures are regional (see Methods) time-window averaged amplitude differences (Target – Non-target) in the early (300-500 ms) and late (500-700 ms) time-windows, and fractional (50%) area latency at Pz for 2-back targets in the full time window (300-700 ms). Values are Pearson’s *r* with *p* values in parentheses. Where quadratic model significantly improved upon linear fit, the associated corrected Akaike Information Criterion (AICc) difference p-value is reported in square brackets

Cells in bold font correspond to those illustrated in scatterplot figures

~ Marginal (p < .1)

*Significant correlation (p < .05) that does not survive FDR correction

**significant correlation (p < .05) that survives FDR correction

^†^quadratic model significantly improved fit

Vineland ABC showed moderate negative correlations with Controls’ P300 amplitude (i.e., higher assessment of general cognition was related to lower P300 amplitude) in 2-back blocks in the left parietal region (early time-window: *r* = -0.51, *p* = 0.049, did not survive FDR; late time-window: *r* = -0.58, *p* = 0.022), and in 1-back blocks in the left frontal region in the late time-window (*r* = -0.61, *p* = 0.015). No ABC-P300 correlations were found for the NF1 group (all |*r*|< 0.3, *p* > 0.3). Sky Search showed moderate negative correlations with NF1s’ P300 amplitude (i.e., higher selective attention performance was related to lower P300 amplitude) in the late time-window in 2-back blocks in the left parietal (*r* = -0.54, *p* = 0.031) and left frontal (*r* = -0.46, *p* = 0.075, did not survive FDR) regions; no such correlations were found in Controls (all |*r*|< 0.4, *p* > 0.15). Sky search also negatively correlated with P300 latency (i.e., higher selective attention performance was related to shorter P300 latencies) in NF1 (*r* = -0.63, *p* = 0.009), but not in Controls (*r* = 0.12, *p* = 0.670). Mean Auditory N-back showed only one moderate (and marginal) negative correlation (i.e., higher auditory N-back performance was related to lower P300 amplitude): Controls’ left frontal amplitude in the late time-window of the 1-back task (*r* = -0.47, *p* = 0.077, did not survive FDR). No significant correlations were found for Conners inattention or hyperactivity measures.

**Fig. 6:**
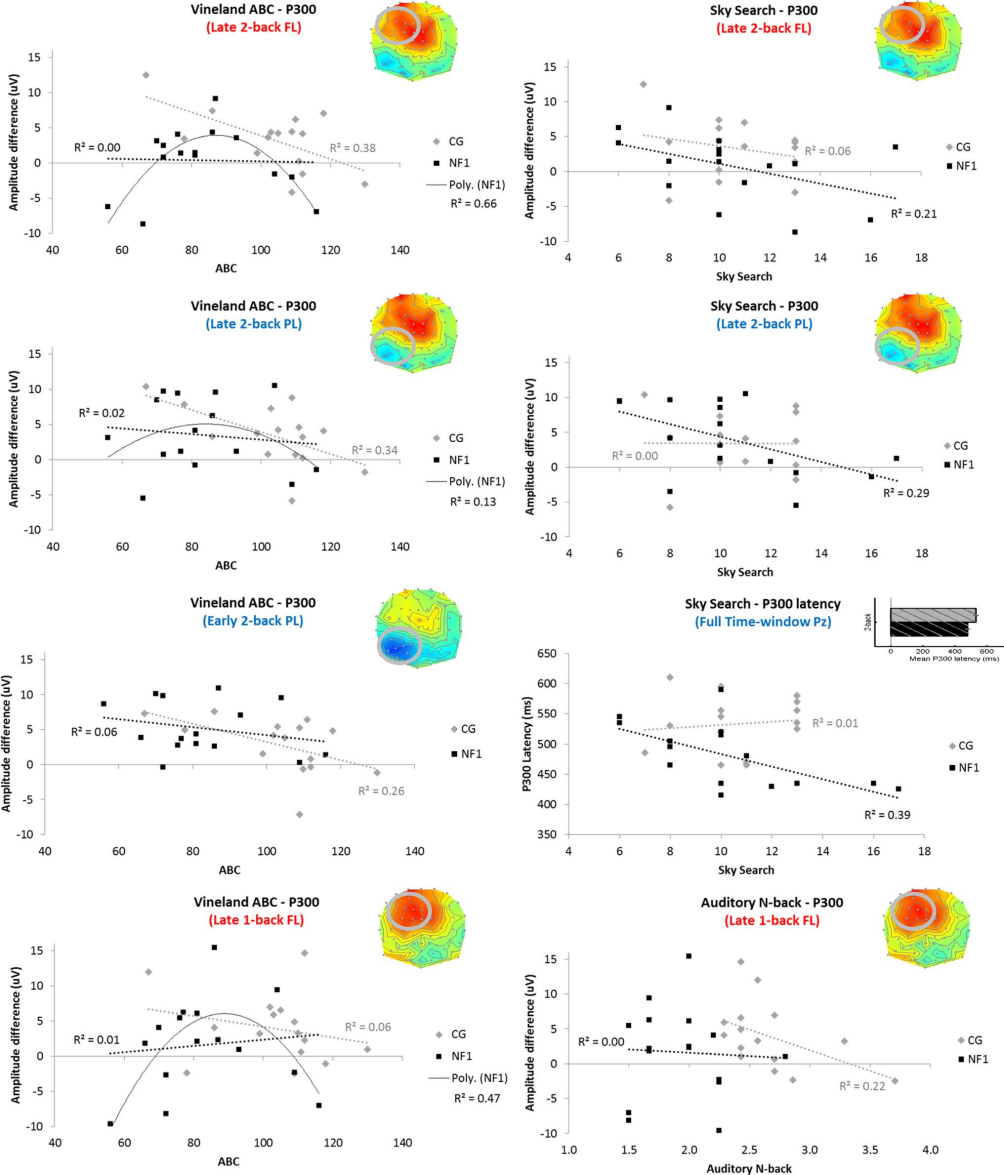
Correlation of behavioural measures (Vineland Adaptive Behaviour Composite, TEACh sky search and auditory n-back) with P300 amplitude (Target-NonTarget difference) and P300 latency (FL: Frontal left, PL: Parietal left) for both groups (CG: Controls; NF1). In each plot, the P300 effect of interest is shown at the top right (with region circled on topographic plots); see Figs. 4 and 5 for details. Colour of sub-title text indicates the direction of the CG-NF1 difference (red: CG>NF1; blue NF1>CG)

Scatterplots of some of these relationships (see Fig. 6) appeared to show a nonlinear inverted-U relationship between P300 amplitude and behavioural measures, particularly in the NF1 group, and in the late time-window. The Matlab function ‘fitglm’ was used to fit generalised linear models to each P300-behavioural pair for each group, with and without a quadratic component, and the Akaike Information Criterion corrected (AICc) was used to test whether the quadratic model was significantly better than the linear model (*p* < 0.05, where *p* = exp((*AIC*_quadratic_ – *AIC*_linear_)/2).

Two significant (and significantly better than linear) quadratic relationships emerged for NF1 ABC and left frontal P300 metrics: late 1-back (quadratic model: *R*^*2*^ = 0.467, *AIC* = 97.74, *p* = 0.023; linear model: *R*^*2*^ = 0.012, *AIC* = 103.82, *p* = 0.697; *p*(quadratic > linear) = 0.048) and late 2-back (quadratic model: *R*^*2*^ = 0.660, *AIC* = 80.44, *p* = 0.002; linear model: *R*^*2*^ = 0.010, *AIC* = 93.43, *p* = 0.911; *p*(quadratic > linear) = 0.002). A quadratic model also improved the R^2^ by more than 10 percentage points for NF1s’ relationship between ABC and left parietal P300 in 1-back late time-window, but this increase was not significant according to AIC, nor was the quadratic model significant (quadratic model: *R*^*2*^ = 0.130, *AIC* = 97.32, *p* = 0.433; linear model: *R*^*2*^ = 0.015, *AIC* = 96.00, *p* = 0.661). In all three cases, the nonlinear relationship was an inverted-U, such that both low and high assessments of general cognitive function were related to relatively low P300 amplitude, whereas mid-range cognition was associated with relatively high P300 amplitude. No other variable combinations showed significant quadratic relationships in either group.

## Discussion

To our knowledge, this is the first study to conduct a detailed assessment of WM and examine the ERP P300 components in NF1. As compared to the controls, the NF1 group showed poorer performance on measures of visuospatial and auditory WM and attentional tasks. As expected, parents reported overall poorer adaptive functioning in the NF1 group as compared to the control group. We found no differences in P300 amplitude at Pz, but shorter latencies in NF1, and topographic differences between the groups that interacted with time-window and memory load: The NF1 group showed reduced left frontal activity, particularly in the late time-window, and greater parietal positivity, particularly in the 2-back task, compared to the control group.

Executive function and WM impairments are core neurocognitive dysfunction in NF1 which impacts considerably on their daily functioning and quality of life. In this study, we used three well established measures of WM tapping into both verbal/auditory and visual domains. Increasing working memory load in the adaptive n-back task differentiated between the groups such that the control group achieved a higher mean n-back level than the NF1 group. The n-back task requires the WM system to be dynamically engaged and involves not only storage and continual updating of information but also interference resolution (Jaeggi et al. 2009). Our findings on the three visual and auditory tasks are convergent and show reduced WM performance in NF1 compared to controls.

Whilst our sample size was limited to draw definitive conclusions, we found the WM improved with age in both groups– a pattern similar to that observed in the general population. However, whilst the control group showed faster RT with age, this pattern was not seen in NF1. Lack of change in RT with age could possibly be attributed to the motor problems previously reported in NF1 which persist with age (Rietman, 2017). An alternative explanation could be due to RT variability – a phenomenon that has been described in ADHD populations (Tamm et al. 2012) and understood to be due to occasional lapses of attention during performance of the cognitive task.

The ERP P300 component was then examined in order to investigate the neural underpinnings of this impairment in working memory. The P300 is strongly associated with memory processes and its amplitude is sensitive to the allocation of processing resources. In healthy subjects, P300 amplitude decreases with increasing memory load (McEvoy et al. 1998), reflecting attentional reallocation. P300 latency is normally considered to be an index of neural speed or efficiency and has been suggested to reflect speed of stimulus classification resulting from discrimination of one event from another. Topographic changes in the spatial distribution of P300 would indicate different relative contributions of neural sources to the scalp-recorded voltages.

In our ‘canonical’ analysis of target P300 amplitude at Pz averaged over the full (300-700 ms) time window, the expected pattern (1-back > 2-back) was found across the whole sample, but there was no interaction between group and memory load, suggesting a similar pattern of P300 amplitude reduction with increased memory load in both NF1 and Controls. However, this was not the full story: P300 latency was found to be shorter in NF1 than in Controls, and here the group x N-back interaction was also significant, with latency being significantly shorter in NF1 than in Controls on the 2-back task. As inspection of the ERP plot at Pz in Fig. 4 shows, there does appear to be a difference in P300 amplitude between groups (NF1 > Controls) in the early portion of the time-window, particularly in the 2-back condition; however, averaging over the whole time-window masked this effect. Given this latency difference, topographic analyses were conducted in early and late portions of the P300 time window, separately. This showed significant differences between the groups in topographic distribution of the P300 amplitude. The NF1 group showed stronger right frontal positivity in the early time window, but weaker left frontal positivity in the late time-window, particularly when the difference between target and non-target P300s was considered. Further, in the 2-back task, the NF1 group showed a stronger mid-to-left parietal positivity, which was also particularly evident on the target–non-target difference.

Topographic differences in P300 are most commonly associated with the distinction between sub-components P3a–which has a more frontal distribution (and presumed frontal cortical sources) and is associated with novelty detection–and P3b–which is parietally maximal (with presumed sources in medial temporal, parietal, and frontal regions) and is associated with task-relevant target detection, allocation of attention, and updating of working memory (Polich, 2007). The pattern of topographic differences found here may therefore reflect dysfunction in both of these systems: Hyperactivity of the target detection/attention system, particularly as load increases (parietal effects), and hypoactivity of parts of the novelty detection system, particularly in the late time window. However, this task was not designed to separate P3a and P3b, as no task-irrelevant infrequent novel stimuli were presented.

A plausible interpretation of the topographic differences between NF1 and controls is that they reflect an imbalance in the distribution of activity across frontal and parietal cortex in service of working memory. Findings in *Nf1*^+/-^ mouse models suggest increased inhibitory activity in the prefrontal cortex and striatum (Goncalves, 2017; Shilyansky et al. 2010) – regions which are critical for working memory performance. In addition, functional MRI studies in NF1 suggest significant hypoactivation of the frontal lobe during reading, visuospatial and working memory tasks (Billingsley et al. 2003, 2004; Ibrahim et al. 2017). Further, diffusion tensor imaging studies suggest widespread white matter microstructural alterations in NF1 which are most pronounced in the frontal lobe (Karlsgodt et al. 2012; Koini et al. 2017). Taken together, the topographic differences seen in this study are in keeping with previous findings of significant hypofrontality in NF1. Coupled with this, we see a differential pattern of topography with overall greater parietal and right hemispheric positivity in the NF1 group as compared to controls. Working memory tasks activate a spatially distributed large-scale network of cortical and subcortical brain regions (Mesulam, 1998). It is possible that differential activation of this network architecture and patterns of interactions between the regions underlie WM impairments in NF1. Indeed, atypical lateralization, specifically reduced left lateralization has been reported in developmental disorders such as ASD and ADHD and thought to reflect impaired task-directed brain system functioning (Hale, 2014; Nielsen, 2014). Future studies could use neural source estimation and/or connectivity analyses to investigate putative changes to these fronto-parietal networks.

A surprising finding in this study was the shorter P300 latency in the NF1 group as compared to the control group, particularly at higher working memory loads (2-back task). P300 latency is thought to reflect stimulus processing time, including recognition and categorization of stimulus. Latency is not always correlated with behavioural response times, since there are many stages of processing between the presentation of a stimulus and the behavioural response, not all of which affect P300 latency (Duncan-Johnson & Donchin, 1980). Indeed in this study we found that although the NF1 group had shorter P300 latencies, there were no group differences in RTs in either the EEG N-back task or the auditory N-back task conducted separately. This dissociation of P300 latency and RT suggests that the latency effects reflect differences in neural processing per se rather than differences in cognitive processing speed or decision latencies. It could be argued therefore that neural processing of stimuli in NF1 is faster but less accurate, possibly contributing to the lower cognitive performance seen on working memory tasks.

To further investigate how P300 components relate to phenotypic presentation, we evaluated five behavioural metrics – behavioural nback performance, attention using a sustainedattention task, Conners inattention and hyperactivity measures, and parent reported adaptive functioning. Correlations between behavioural and P300 measures showed different patterns in Controls and NF1. In the NF1 group, Sky Search, a measure of selective attention, was negatively correlated with P300 amplitude difference (Target – Non-target) in the late time-window in the 2-back task, in both left parietal and left frontal (the latter only marginally) regions. This suggests engagement of these neural resources either compensates for or contributes to low selective attention abilities in NF1. Sky Search was also negatively correlated with 2-back P300 latency in NF1 (only); that is, P300 reached its peak faster in NF1 participants with higher selective attention abilities. It is tempting to conclude that this effect is due to an association between selective attention and decision time; however, it is worth noting that only NF1 participants with the *lowest* selective attention abilities have P300 latencies in the Control range, and those with the *highest* attention abilities have unusually low P300 latencies, out of the range of Controls. By contrast, Sky Search and Conners measures showed no significant correlations with Controls’ P300 amplitude or latency. These findings should be studied in more detail in the future studies.

In Controls, Vineland ABC, a measure of adaptive functioning, was negatively correlated with several P300 amplitude difference measures, all in 2-back blocks: the left parietal region in both early (which did not survive FDR correction) and late time-windows, and the left frontal region in the late time-window. These negative correlations suggest that the underlying neural resources are engaged more by those Controls with lower adaptive functioning, perhaps as a compensatory strategy. By contrast, none of these linear correlations were significant in the NF1 group; however, several adaptive function-P300 amplitude relationships were found to show a significant non-linear, inverted-U relationship in the NF1 group: late time-window left frontal activity in both 1- and 2-back blocks. This pattern suggests that NF1 individuals with mid-range adaptive function are able to engage this compensatory mechanism in order to maintain task performance, but that NF1 individuals with the lowest adaptive function fail to engage this mechanism (hence the down-turn in amplitude at the lower end of the ABC range). A similar, but weaker, pattern was seen in NF1s’ late left parietal activity in 2-back blocks, but this quadratic relationship was not significant.

Our findings thus expand our understanding of the neural correlates of altered of WM deficits in NF1. Cognitive and behavioural impairments remain a primary cause of morbidity in the NF1 population (Hyman et al. 2005). Whilst the discovery of pharmacological agents that could reverse the underlying deficits is important, understanding the underlying neurobiology in humans will be important for successful translational of basic science research in animal models. Research over the last decade has demonstrated the challenges of successful translation of preclinical findings into clinical trials no doubt in part due to overreliance on cognitive end-points which lack sensitivity (Payne, 2019). The results of this study suggest that EEG may provide an unbiased biomarker of cortical function in NF1. Limitations of our study include the relatively small sample size of the cohort. However, given that NF1 is a rare genetic condition it is more challenging to recruit larger sample sizes in a single centre study. As both P300 latency and amplitude index different aspects of brain maturation, it may be important to understand the P300 changes across the developmental life span in NF1.

In summary, our findings suggest impaired WM performance in the NF1 group replicating previous findings. We investigated for the first time the P300 ERP components of WM in NF1 and find topographic differences in the P300 amplitude in the NF1 group. Our findings offer further insights into how NF1 may influence cognitive processing abilities, but larger studies are needed to understand how the findings reported in this paper relate to the learning and memory deficits common to the NF1 population.

## Supplementary Information

Below is the link to the electronic supplementary material.

Supplementary file1 (DOCX 23 kb)
