# Supplementary material for: Cognitive and Electrophysiological Correlates of Working Memory Impairments in Neurofibromatosis Type 1
Source: J Autism Dev Disord. 2021 May 8;52(4):1478–94. doi: 10.1007/s10803-021-05043-3 (PMC8938373; doi:10.1007/s10803-021-05043-3)
Supplement: Supplementary file 1 — Supplementary file1 (DOCX 23 kb) [file 10803_2021_5043_MOESM1_ESM.docx]

**Supplementary tables**

Supplemental Table 1: Pearson’s *r* with corresponding *p* values between age and cognitive performance of NF1 and Control participants.

|  |  |  | | NF1 |  |  | Controls | |  | NF1 - Control |
| --- | --- | --- | --- | --- | --- | --- | --- | --- | --- | --- |
| Task | Modality | | DV | r | *p* | | r | *p* | | *p* |
| N-back | Visual | | 1-back RT | -.114 | *.675* | | -.302 | *.256* | | *.615* |
|  |  | | 2-back RT | .062 | *.820* | | -.631 | *.009* | | *.****040**** |
|  |  | | mean nback | .369 | *.159* | | -.119 | *.661* | | *.196* |
|  | Auditory | | 1-back RT | .317 | *.232* | | -.299 | *.260* | | *.105* |
|  |  | | 2-back RT | .078 | *.773* | | -.301 | *.257* | | *.321* |
|  |  | | mean nback | .487 | *.055* | | .443 | *.085* | | *.886* |
| Corsi | Visual | | span | .577 | *.019* | | .433 | *.094* | | *.620* |

Supplementary Table 2. Correlations between age and behavioural performance on the EEG N-back task.

|  |  | NF1 |  |  | Control |  |  | NF1 - Control |
| --- | --- | --- | --- | --- | --- | --- | --- | --- |
| Behavioural  DV (EEG N-back task) | N-back | r | *p* | | r | *p* | | *p* |
| Hits-False Alarms | 1 | .493 | .052 | | .328 | .233 | | .618 |
|  | 2 | .524 | **.037*** | | .455 | .089 | | .820 |
|  |  |  |  | |  |  | |  |
| RT | 1 | -.295 | .267 | | -.387 | .155 | | .795 |
|  | 2 | -.138 | .609 | | -.520 | **.047*** | | .275 |

Notes: DV=dependent variable: RT=response time; statistics are Pearson’s correlation and associated p-value.

Supplementary Table 3. Correlations between age and P300.

|  |  |  |  |  | NF1 |  |  | Control |  |  | NF1 - Control |
| --- | --- | --- | --- | --- | --- | --- | --- | --- | --- | --- | --- |
| P300  DV | Time-win | Cond | N-back | Elec | r | *p* | | r | *p* | | *p* |
| FAL | full | T | 1 | Pz | -.234 | *.402* | | -.148 | *.598* | | *.828* |
|  |  |  | 2 | Pz | -.262 | *.327* | | -.466 | *.080* | | *.555* |
|  |  |  |  |  |  |  | |  |  | |  |
| amp | full | T | 1 | Pz | .148 | *.599* | | .033 | *.908* | | *.775* |
|  |  |  | 2 | Pz |  |  | |  |  | |  |
|  |  |  |  |  |  |  | |  |  | |  |
| amp | early | T | 1 | FL | -.250 | *.351* | | -.109 | *.700* | | *.715* |
|  |  |  |  | FR | -.178 | *.508* | | -.020 | *.945* | | *.688* |
|  |  |  |  | PL | .108 | *.690* | | -.068 | *.809* | | *.659* |
|  |  |  |  | PR | .043 | *.875* | | -.009 | *.973* | | *.896* |
|  |  |  | 2 | FL | -.074 | *.785* | | .304 | *.270* | | *.332* |
|  |  |  |  | FR | -.019 | *.944* | | .422 | *.117* | | *.241* |
|  |  |  |  | PL | -.008 | *.976* | | .237 | *.395* | | *.533* |
|  |  |  |  | PR | -.038 | *.888* | | .136 | *.629* | | *.662* |
|  |  | NT | 1 | FL | .067 | *.807* | | -.169 | *.548* | | *.554* |
|  |  |  |  | FR | .132 | *.627* | | -.209 | *.455* | | *.390* |
|  |  |  |  | PL | .250 | *.350* | | -.236 | *.396* | | *.215* |
|  |  |  |  | PR | .039 | *.885* | | -.431 | *.108* | | *.211* |
|  |  |  | 2 | FL | .021 | *.939* | | .367 | *.179* | | *.363* |
|  |  |  |  | FR | -.140 | *.606* | | .293 | *.289* | | *.269* |
|  |  |  |  | PL | .131 | *.628* | | .164 | *.560* | | *.934* |
|  |  |  |  | PR | -.143 | *.598* | | -.093 | *.741* | | *.900* |
|  | late | T | 1 | FL | -.159 | *.558* | | -.532 | ***.041**** | | *.279* |
|  |  |  |  | FR | -.181 | *.503* | | -.415 | *.124* | | *.517* |
|  |  |  |  | PL | .191 | *.478* | | -.293 | *.289* | | *.216* |
|  |  |  |  | PR | .009 | *.972* | | -.177 | *.528* | | *.638* |
|  |  |  | 2 | FL | .097 | *.721* | | .001 | *.999* | | *.809* |
|  |  |  |  | FR | .055 | *.840* | | .060 | *.831* | | *.989* |
|  |  |  |  | PL | .018 | *.947* | | .072 | *.798* | | *.892* |
|  |  |  |  | PR | -.015 | *.956* | | .033 | *.908* | | *.905* |
|  |  | NT | 1 | FL | .044 | *.872* | | -.332 | *.227* | | *.332* |
|  |  |  |  | FR | .056 | *.837* | | -.378 | *.165* | | *.257* |
|  |  |  |  | PL | .193 | *.473* | | -.368 | *.177* | | *.146* |
|  |  |  |  | PR | .008 | *.978* | | -.558 | ***.030**** | | *.111* |
|  |  |  | 2 | FL | .030 | *.913* | | -.049 | *.861* | | *.843* |
|  |  |  |  | FR | -.136 | *.616* | | -.072 | *.799* | | *.872* |
|  |  |  |  | PL | .248 | *.355* | | .037 | *.895* | | *.590* |
|  |  |  |  | PR | .033 | *.905* | | -.100 | *.724* | | *.741* |

Notes: DV=dependent variable: amp=amplitude, FAL=fractional area latency; Time-win=time-window: full=300-700ms, early=300-500ms, late=500-700ms; Cond=stimulus condition: T=target, NT=non-target; Elec=electrode clusters: F=frontal, P=parietal, L=left, R=right; statistics are Pearson’s correlation and associated p-value.
